# LysGH15 kills *Staphylococcus aureus* without being affected by the humoral immune response or inducing inflammation

**DOI:** 10.1038/srep29344

**Published:** 2016-07-07

**Authors:** Lei Zhang, Dong Li, Xinwei Li, Liyuan Hu, Mengjun Cheng, Feifei Xia, Pengjuan Gong, Bin Wang, Jinli Ge, Hao Zhang, Ruopeng Cai, Yanmei Wang, Changjiang Sun, Xin Feng, Liancheng Lei, Wenyu Han, Jingmin Gu

**Affiliations:** 1 College of Veterinary Medicine, Jilin University, Changchun 130062, P. R. China; 2 Department of Immunology, College of Basic Medical Sciences, Jilin University, Changchun, 130021, P. R. China; 3 Jiangsu Co-innovation Center for the Prevention and Control of important Animal Infectious Disease and Zoonoses, Yangzhou 225009, P. R. China

## Abstract

The lysin LysGH15, derived from the staphylococcal phage GH15, exhibits a wide lytic spectrum and highly efficient lytic activity against methicillin-resistant *Staphylococcus aureus* (MRSA). Here, we found that LysGH15 did not induce resistance in MRSA or methicillin-sensitive *S. aureus* (MSSA) strains after repeated treatment. Although LysGH15 triggered the generation of LysGH15-specific antibodies in mice, these antibodies did not block lytic activity *in vitro* (nor the binding capacity of LysGH15). More importantly, when the antibody titre was highest in mice immunized with LysGH15, a single intravenous injection of LysGH15 was sufficient to protect mice against lethal infection with MRSA. These results indicated that LysGH15-specific antibodies did not affect the killing efficiency of LysGH15 against MRSA *in vitro* or *in vivo*. LysGH15 also reduced pro-inflammatory cytokines in mice with lethal infections. Furthermore, a high-dose LysGH15 injection did not cause significant adverse effects or pathological changes in the main organs of treated animals. These results provide further evidence for the administration of LysGH15 as an alternative strategy for the treatment of infections caused by MRSA.

*Staphylococcus aureus* is a ubiquitous and zoonotic pathogen that causes high morbidity and mortality in a variety of diseases. Infections caused by *S. aureus* are a major health problem in both hospital and community settings^1^,^2^. The treatment of these infections has become increasingly difficult because of the emergence of multidrug-resistant strains, particularly methicillin-resistant *S. aureus* (MRSA), during the past decade^3^,^4^,^5^. Therefore, there is an urgent need for novel therapeutic agents that act directly against this formidable pathogen^6^,^7^,^8^.

Bacteriophage endolysin is encoded by the bacteriophage genome and is synthesized at the end of the phage lytic life cycle to lyse the host cell^9^. Lysin typically accesses and cleaves cell wall peptidoglycans when added exogenously, lysing cells within seconds to minutes after contact through hypotonic lysis^10^. It has been reported that staphylococcal phage lysins cleave the sites between D-alanine of the stem peptide and glycine of the cross-bridge peptide and possess N-acetylmuramoyl-L-alanine amidase activity^11^. Several lysins have been successfully used as tools to destroy the cell wall of pathogenic bacteria, such as *Streptococcus pyogenes*^12^, *S. pneumoniae*^13^ and *Bacillus anthracis*^14^. Thus, phage lysins have been suggested as a promising alternative to antibiotics for the treatment of bacterial infections^10^,^15^.

LysGH15, a lysin derived from the staphylococcal phage GH15, demonstrated strong lytic activity against MRSA both *in vitro* (>5-fold reduction over 30 min) and *in vivo* (>2-fold reduction over 2.5 h)^16^,^17^,^18^,^19^. In addition, to explore the molecular mechanism of this lytic activity, the structures of three individual domains of LysGH15 were also determined^19^. However, this phage lysin could be an immunogenic protein, and its use is likely to induce an immune response^20^,^21^. Whether specific anti-LysGH15 antibodies could block the activity of LysGH15 or even cause inflammatory disease remains unknown. Therefore, the current study addressed whether *S. aureus* develops resistance against LysGH15, similar to antibiotics, and how the host immune system interacts with LysGH15.

## Results

### *S. aureus* did not develop resistance after repeated exposure to LysGH15

**Figure 1:**
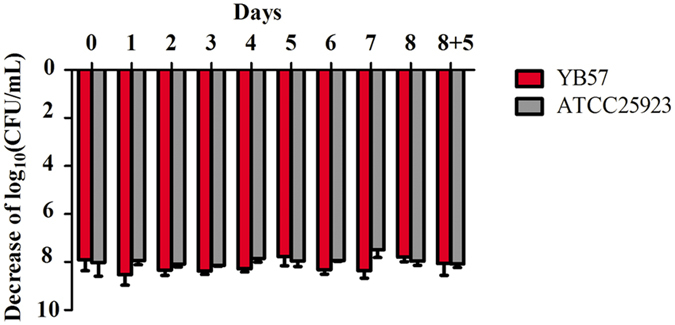
The sensitivity of different generation cells to LysGH15. The CFU/mL descent was used to evaluate the antibacterial activity of LysGH15 (50 μg/mL) on different generation cells (10^10^ CFU/mL). The values represent the mean ± SD (n = 3).

The minimum inhibitory concentration (MIC) values of LysGH15 for the MRSA (YB57) and methicillin-sensitive *S. aureus* (MSSA) (ATCC25923) strains were 15.625 and 31.25 μg/mL, respectively. Both the MRSA (YB57) and MSSA (ATCC25923) strains were analysed for the development of resistance to LysGH15 using plate lysis and MIC assays. When *S. aureus* was exposed to serial dilutions of LysGH15, no spontaneous resistant mutants (neither the MRSA (YB57) nor the MSSA (ATCC25923) strains) were recovered. The cells from different passages showed similar sensitivity to LysGH15 (Fig. 1). Additionally, the MICs of cells obtained in each passage showed the same values as original bacteria (Data not shown).

### Anti-LysGH15 antibody titres in serum

**Figure 2:**
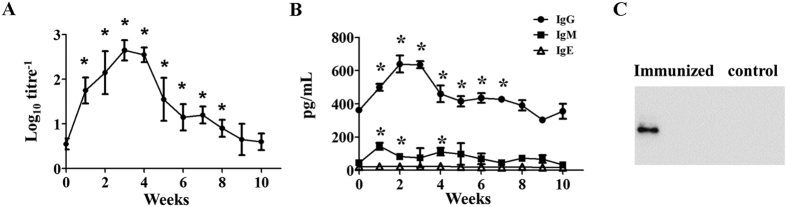
LysGH15 induced specific IgG antibodies. Serum samples from immunized mice and control mice were collected every week for 10 weeks, and the titres (**A**) and concentrations of IgG, IgM and IgE isotypes (**B**) were measured using ELISA. (**C**) Western blotting assay of LysGH15. Mouse serum at 3 weeks post-immunization was used as the primary antibody (dilution 1:500), and HRP-labeled anti-mouse IgG antibody was used as the secondary antibody. The left lane shows the results for the LysGH15-injected mouse. The right lane shows the LysGH15 buffer-injected mouse. (n = 6 mice per group per experiment). *P < 0.05 compared with unimmunized mice. The data are representative of three experiments.

Serum was collected every week over a 10-week period from mice (n = 6) injected subcutaneously (s.c.) with LysGH15 (50 μg). Enzyme-linked immunosorbent assay (ELISA) analysis demonstrated that anti-LysGH15 antibodies were detectable at 1 week post-LysGH15 administration, peaking at 3 weeks with a log10titre^−1^ of 2.7; the presence of LysGH15-specific IgG antibodies was also confirmed through Western blot analysis (Fig. 2A,C). The primary antibody isotype was IgG (Fig. 2B).

### The activity of LysGH15 was not affected by anti-LysGH15 serum

**Figure 3:**
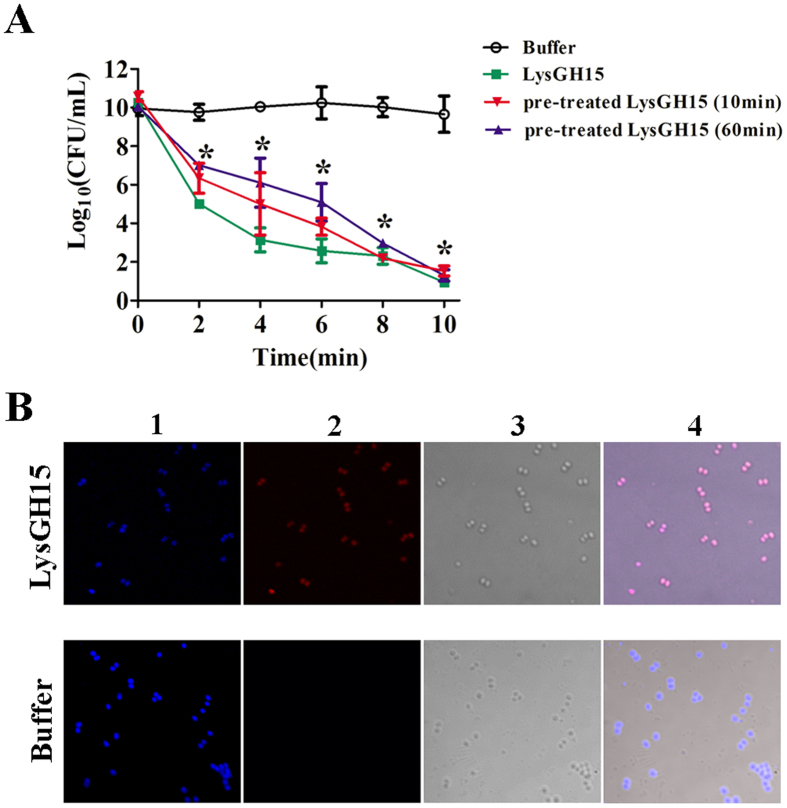
Anti-LysGH15 serum did not neutralize the activity of LysGH15 *in vitro*. (**A**) The influence of anti-LysGH15 serum on the lytic activity of LysGH15. LysGH15 was pre-incubated with serum (dilution 1:500) from immunized mice for 10 min or 1 h, and this mixture or LysGH15 alone or control buffer was added to cultures of the MRSA strain (YB57). CFU numbers were counted at different time points as indicated. (n = 3 per group per experiment). *P < 0.05 compared with the buffer control. The data are representative of 3 experiments. (**B**) The influence of anti-LysGH15 serum on the binding activity of C54S-LysGH15. YB57 cells were dyed with Hoechst No. 33342 fluorescent dye, and then C54S-LysGH15 or buffer was pre-incubated with serum (dilution 1:500) prior to the addition to YB57. TRITC-conjugated goat anti-mouse IgG was used. The images were acquired using laser scanning confocal microscopy (LSCM) as described in the Methods section. 1. Localization at 405 nm (blue fluorescence, emitted from Hoechst No. 33342 fluorescent dye). 2. Localization at 543 nm (red fluorescence, emitted from TRITC-conjugated Goat-anti-mouse IgG). 3. Image of an ordinary ray (normal light). 4. Overlay of the pictures shown in 1, 2, and 3.

To test the effects of anti-LysGH15 serum on the bactericidal activities of LysGH15, serum was collected 3 weeks after s.c. immunization with LysGH15 (50 μg); this time point showed the highest anti-LysGH15 antibody titre. LysGH15 was incubated with the serum for 10 min and was subsequently added to the cultured MRSA strain YB57. As shown in Fig. 3A, the colony count decreased 5.3 log units within 2 min after treatment with serum-incubated LysGH15. However, the bactericidal activity of LysGH15 treated with LysGH15-immunized serum showed no significant difference compared to LysGH15 treated with normal mouse serum. The bactericidal activity of LysGH15 was not affected even after incubation with anti-LysGH15-serum for a longer time (60 min).

Next, to detect whether the anti-LysGH15 serum affected the binding between the lysin and the MRSA strain YB57, a modified LysGH15, C54S-LysGH15 (deficient in lytic activity, but with maintained binding activity), was used. Red fluorescence on YB57 was observed when the bacteria were incubated with C54S-LysGH15 pre-incubated with serum. As a control, red fluorescence was not detected on YB57 when the bacteria were incubated with the anti-LysGH15 serum alone, suggesting that anti-LysGH15 antibodies bind to C54S-LysGH15 but do not block the binding between C54S-LysGH15 and YB57 (Fig. 3B).

### LysGH15 protected LysGH15-immunized mice against lethal infection with *S. aureus*

**Figure 4:**
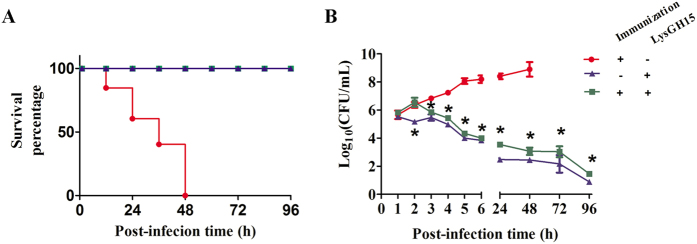
LysGH15 protected the mice from lethal MRSA infection. (**A**) The rescue of mice from lethal MRSA (YB57) infection using LysGH15. BALB/c mice immunized with lysin or control buffer were challenged intravenously with YB57 MRSA (1 × 10^10^ CFU/mouse). One hour later, 50 μg of LysGH15 or control buffer was injected into the mice. (**B**) Colony counts in peripheral blood samples. At the indicated times, the bacterial counts (CFU/mL) in the mice were determined from peripheral blood samples (10 μL) obtained from the caudal vein (n = 6 mice per group per experiment). *P < 0.05 compared with the buffer-treated control. The data are representative of 3 experiments.

**Figure 5:**
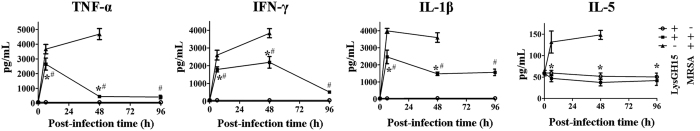
LysGH15 reduced pro-inflammatory cytokines. The BALB/c mice immunized with LysGH15 were injected intravenously with MRSA strain YB57 (1 × 10^10^ CFU/mouse). One hour later, these mice were treated with LysGH15 or the control buffer. At the indicated time points, the levels of TNF-α, IFN-γ, IL-1β, and IL-5 in the serum were determined (n = 6 mice per group per experiment). *P < 0.05 compared with the buffer-treated control; ^#^P < 0.05 compared with the uninfected control. The data are representative of 3 experiments.

In animal experiments, a single intravenous injection of LysGH15 (50 μg) administered 1 h after MRSA infection at double the minimum lethal dose was sufficient to protect the mice (Fig. 4A). We further observed that even when mice were immunized with LysGH15 (50 μg) 3 weeks prior, treatment with LysGH15 (50 μg) 1 h after bacterial challenge led to significantly reduced bacteremia; in contrast, no mice in the untreated group survived more than 48 h (Fig. 4A). The amount of bacteria in the mouse blood was also analysed. The immunized mice treated with LysGH15 demonstrated a colony-forming unit (CFU) reduction of 1.8 log units at 6 h after treatment. The final CFU count was significantly lower than that of the no-treatment group (CFU increased to 8.9 log units) and similar to that of the unimmunized group treated with LysGH15 alone (Fig. 4B). The elimination of *S. aureus* (YB57) through LysGH15 was not affected by whether the mice were immunized with LysGH15. Furthermore, pro-inflammatory cytokines (TNF-α, IFN-γ, IL-1β and IL-5) induced in response to MRSA infection were significantly decreased after LysGH15 treatment (Fig. 5).

### A high dose of LysGH15 induced no significant side effects *in vivo*

**Figure 6:**
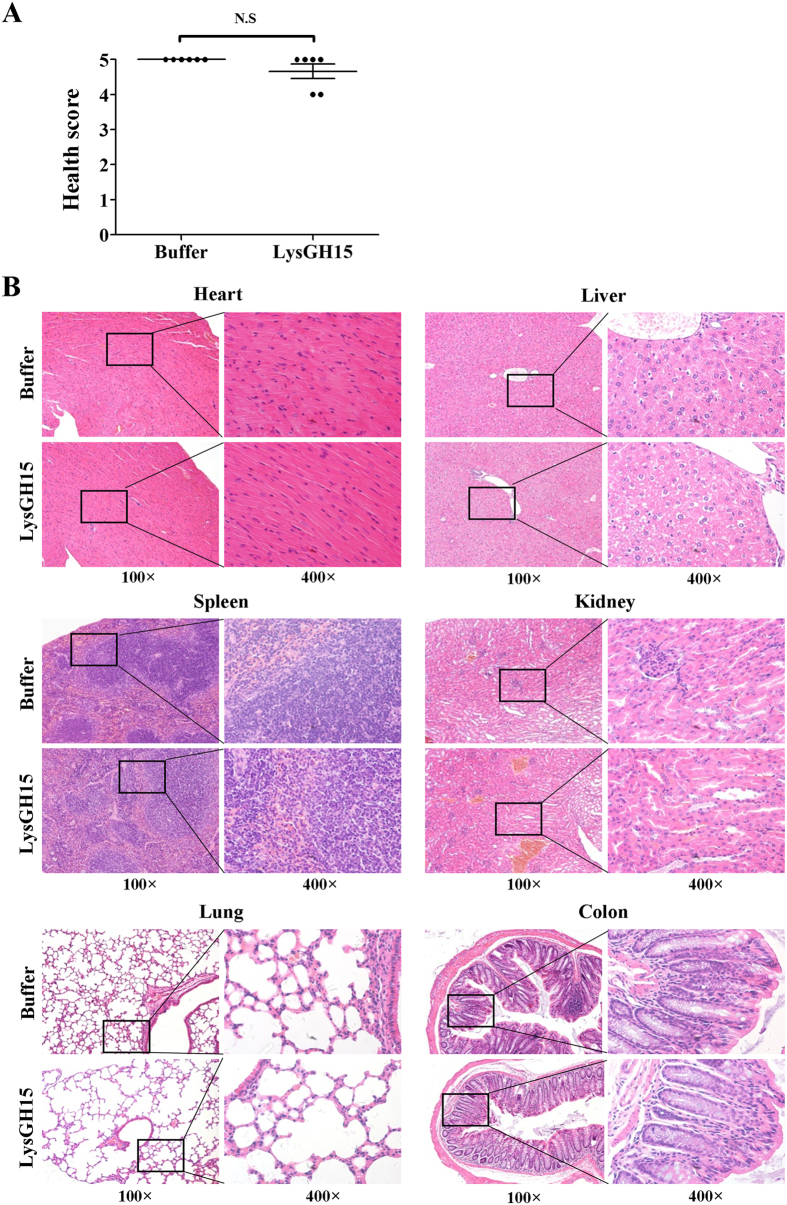
LysGH15 did not cause significant adverse effects. The mice were immunized with LysGH15 at 3 weeks prior to the intravenous administration of large amounts of LysGH15 or buffer. The mice were monitored for 10 days and euthanized on day 11. (**A**) The health score was assessed as described in the Methods section. (**B**) Pathological changes and histopathology of the organs. The hearts, livers, spleens, lungs, kidneys, and colons were stained with haematoxylin and eosin. n = 6 mice per group per experiment. *P < 0.05 compared to the buffer-treated control; N.S. not significant. The data are representative of three experiments.

The potential side effects of LysGH15 were investigated. First, the mice were immunized through s.c. injection of LysGH15 (50 μg). After 3 weeks, 10 mg of LysGH15 was intravenously injected into the immunized mice. We observed no side effects, based on the aspects and behaviours of the treated mice observed for 10 days (Fig. 6A). Moreover, the tissues harvested from immunized mice treated with large amounts of LysGH15 showed no severe inflammation or other pathological changes, as shown in Fig. 6B.

## Discussion

Lysins are highly evolved enzymes derived from phages that lyse the bacterial cell wall. In Gram-positive bacteria, small quantities of lysin added externally are sufficient to induce the log-fold death of the target bacterium. The advantages of lysin over conventional antibiotics include specificity for the pathogen without disturbing the normal flora, the low chance of bacterial resistance to lysins, and the killing of pathogens colonizing mucosal surfaces, a previously unavailable capacity^22^. Thus, based on these virtues, phage lysins show potential for the treatment of bacterial infections caused by antibiotic-resistant strains.

Bacterial resistance is the most serious concern in antibacterial chemotherapy. Bacterial resistance frequently develops as a result of the high adaptive capacity of these microorganisms, as bacteria are prone to genetic changes and the acquisition of mobile genetic elements through horizontal gene transfer (HGT)^23^. Recent findings have suggested that the largest extent of the horizontal transfer of resistance plasmids in the environment occurs in the presence of stressors, such as antibiotics and some nanomaterials at extremely low, sublethal concentrations^24^,^25^,^26^. In contrast, there is a low chance of bacterial resistance to lysin. In the present study, we also showed that LysGH15-resistant strains did not develop after repeated exposure, consistent with previous studies of phage lysins, such as Clys and PlySs2^27^,^28^. The low likelihood of bacterial resistance to lysins might reflect the manner in which phage lysins have evolved: the binding domains of the lysins tightly bind to a critical component in the bacterial cell wall, and the binding target is typically difficult for the bacteria to reverse^29^. Indeed, any bacterial mutations that avoid this type of attack would also affect the proliferation of the bacterium itself^14^.

We observed that LysGH15 was indeed an immunogenic protein that could induce specific antibodies as confirmed through ELISA, Western blotting and immunofluorescence assays. Nonetheless, specific anti-LysGH15 antibodies were unable to neutralize the binding and lytic activity of LysGH15. LysGH15 is not a unique lysin in terms of this trait. To our knowledge, other phage lysins including Clys^21^, Cpl-1^30^, MV-L^31^ and PlySs2^27^ are not significantly inactivated by immunized serum. In a previous study, we observed that the N-terminal CHAP domain and C-terminal SH3b domain are essential for the activities of LysGH15, and these regions contribute to grooves comprising key residues^16^. Thus, anti-LysGH15 antibodies might not block the grooves of the CHAP and SH3b domains. In addition, this phenomenon might also reflect the fact that the affinity of cell wall-lysin binding might be higher than the affinity of antibody-lysin binding^32^.

The *in vivo* experiments showed that repeated infusions of LysGH15 could also efficiently protect mice against lethal MRSA infections. Moreover, the levels of pro-inflammatory cytokines were significantly decreased in infected mice after treatment with LysGH15. Recent studies have shown that pro-inflammatory cytokines play a critical role in the pathogenesis of MRSA infections^29^,^33^,^34^. Notably, LysGH15 does not enhance IgE levels among total serum antibodies. IgE activates mast cells and basophils via binding to FcεR on the cell membrane, and these cells release histamines, vasoactive mediators and pro-inflammatory cytokines upon activation, an essential step during asthma and most allergic reactions^35^,^36^. Furthermore, histological analysis showed that neither repeated injections nor large-dose infusions of LysGH15 resulted in inflammation or mast cell activation in major organs. Notably, clinical trials will provide the final answers to whether the human antibodies produced by LysGH15-treated patients neutralize the lytic efficacy of this lysin. In particular, the potential for an IgE response in humans should be investigated, as this effect would trigger severe side effects. Together, our results provide further evidence that the administration of LysGH15 might be an alternative strategy for the treatment of MRSA infections.

## Materials and Methods

### Ethics statement

Female BALB/c mice weighing 20 to 22 g (purchased from the Experimental Animal Centre of Jilin University, Changchun, China) were housed in filter-top cages in an air-conditioned animal facility in the National Experimental Teaching Demonstration Centre of Jilin University (Changchun, China). Water and normal mouse chow were provided *ad libitum*, and the mice were monitored daily. All animal experimental procedures were performed in strict accordance with the Regulations for the Administration of Affairs Concerning Experimental Animals approved through the State Council of People’s Republic of China (1988.11.1) and with approval of the Animal Welfare and Research Ethics Committee at Jilin University.

### Bacterial strains

MRSA strain YB57 and MSSA strain ATCC25923 were maintained in the laboratory^16^,^17^,^19^ and used throughout the experimental period. YB57 and ATCC25923 were routinely grown in brain heart infusion (BHI) broth (BD Biosciences, CA, US) at 37 °C with shaking at 200 revolutions per minute (rpm).

### Purification of LysGH15

An *Escherichia coli* BL21(DE3) strain expressing the full-length LysGH15 protein was previously constructed in the laboratory, and LysGH15 was expressed and purified as described in a previous report^16^,^19^.

### Determination of MIC

The protocol of Wiegand *et al*. was used to determine the MIC^37^. Briefly, YB57 or ATCC25923 cells were suspended in BHI (5 × 10^5^ CFU/mL) and subsequently distributed to each well of a 96-well microplate. A titration (500 μg/mL to 0.244 μg/mL) of LysGH15 or control buffer was used to challenge the cells. The MIC values were determined through the detection of cell pellet formation after incubation for 48 h at 37 °C. The cell pellet was obtained after centrifugation (5,000 × *g*; 10 min; 4 °C), and the vital cells in the pellet were detected using the Alamar Blue assay^27^.

### Determination of bacterial resistance to LysGH15

Resistant development was tested using repeated exposures in both plate lysis and MIC assays as previously described, with some modifications^38^. For the plate lysis assay, 2-fold serial dilutions of the proteins were spotted (10 μL) onto a freshly plated lawn of MRSA (YB57) or MSSA (ATCC25923) on BHI plates and grown overnight at 37 °C. LysGH15 dilutions were made in phosphate-buffered saline (PBS) buffer (137 mM NaCl, 2.7 mM KCl, 50 mM Na_2_HPO_4_, and 10 mM KH_2_PO_4_, pH 7.4), starting at 54 μg/mL. The cells from spots with a not fully cleared lawn (sub-lethal) were scraped, inoculated in 5 mL of BHI broth and grown to mid log phase (OD_600_ nm 0.4–0.6) to generate a new lawn for the next round of plating and LysGH15 exposure. After 8 rounds of exposure, the cells were grown for 5 additional overnight cultures on BHI plates in the absence of LysGH15. A new plate lysis assay was performed with the cultures resulting from these 5 non-selective grow-outs to re-assess the sensitivity of the putative resistant cultures to LysGH15. Cells obtained from each generation were used to detect the sensitivity to LysGH15, and the MICs of these cells were also determined.

### Mouse immunization model

The mice were immunized s.c. with LysGH15 (50 μg) or control buffer^17^,^18^. After immunization, the blood samples were collected weekly for 10 weeks for serological analyses. The collected blood samples were centrifuged (5,000 × *g*; 10 min; 4 °C), and then the serum was stored at −20 °C.

### Enzyme-linked immunosorbent assay

An indirect ELISA was used to measure the titres of total LysGH15-specific antibodies. Briefly, a 96-well ELISA plate (JET BIOFIL, China) was coated with 100 μL of purified His-LysGH15 (5 μg/mL) in 0.05 M carbonate-bicarbonate buffer (pH = 9.6) overnight at 4 °C and blocked for 2 h at 37 °C with 5% skim milk. The plates were incubated with PBS alone or with 100 μL of mouse plasma samples diluted 20-fold using PBS for 1–2 h at room temperature. After washing, the plates were incubated with 100 μL of goat anti-mouse IgG (1:1,000 dilution in 1% PBS) for 1 h. After washing again, 100 μL of TMB substrate solution was added to the plates. The IgG, IgM and IgE titres of the sera were measured using ELISA (R&D Systems) for 10 weeks, according to the manufacturer’s instructions. The OD_450_ value was measured using an ELISA platereader^39^,^40^. The antibody titre was defined as the reciprocal of the dilution yielding absorption at 450 nm.

The concentrations of the cytokines in mouse serum samples were quantified using ELISA (eBioscience, CA, US), according to the manufacturer’s instructions^34^.

### Western blotting analysis

Blood samples from mice immunized or unimmunized with LysGH15 were collected after 3 weeks for the detection of antibodies through Western blotting. Purified LysGH15 protein fractionated using sodium dodecyl sulphate polyacrylamide gel electrophoresis (SDS-PAGE) was transferred to a polyvinylidene fluoride membrane (Millipore, MA, US). After blocking with 5% skim milk in PBS containing 0.05% Tween 20 (PBST), the membrane was incubated with the serum collected at 3 weeks at a working dilution of 1:500 in PBST for 1 h at room temperature. The membrane was subsequently incubated with HRP-conjugated goat anti-mouse IgG (Bio-Rad Laboratories, CA, US) diluted 1:5,000 in PBST. After rinsing, the enzymatic reaction was developed using Millipore Immobilon Western Chemiluminescent HRP Substrate (Millipore, MA, US)^39^,^40^,^41^.

### Catalytic activity assay of LysGH15

The MRSA strain YB57 was grown to the exponential growth phase (an OD_600_ nm value of 0.6) in BHI broth at 37 °C with shaking at 200 rpm. The bacteria were collected and washed three times (5000 × *g* for 1 min at 4 °C) with PBS. To determine whether antibodies could interfere with the catalytic activity of LysGH15, a neutralization assay was performed, mixing 20 μL (500 μg/mL) of the LysGH15 solution with 80 μL of serum collected from LysGH15-immunized or LysGH15-unimmunized mice (the dilution was 1:500), followed by incubation at 37 °C for 10 min or 1 h^13^,^31^. Subsequently, the mixture was added to YB57 (100 μL, 1 × 10^10^ CFU/mL) and further incubated at 37 °C. The CFU values were calculated at various time points (2, 4, 6, 8, and 10 min) for the mice in each group.

### Immunofluorescence assay

A 1-mL aliquot of stationary phase YB57 was collected and plated onto 35-mm glass bottom dishes (NEST, USA), washed with PBS and fixed in 4% paraformaldehyde (4 °C) for 15 min. Subsequently, the bacteria were washed three times (5 min per time) with slight shaking, followed by blocking with 200 μL of 5% bovine serum albumin in PBS for 30 min at 37 °C. After blocking, the bacteria were dyed with 20 μmol∙L^−1^ Hoechst No. 33342 fluorescent dye for 10 min at 37 °C and washed five times with PBS. The mouse-anti-LysGH15 sera (1:500 dilution, 100 μL) and C54S-LysGH15 (produced in our lab, residue C54 was mutated to serine; the C54S mutation in the full-length LysGH15 results in the complete loss of lytic activity) were incubated at 37 °C for 10 min^18^. Mixtures or single-mouse anti-LysGH15 serum samples were incubated with the dyed YB57 for 30 min at 37 °C^19^. Subsequently, the treated YB57 was washed three times (5 min per wash) and incubated with 100 μL of TRITC-conjugated goat anti-mouse IgG (1:1,000) (Abbkine, CA, US) for 30 min at 25 °C. After incubation, the cells were washed five times with PBS^42^. Laser scanning confocal microscopy (LSCM) was used to detect the fluorescence of the treated cells irradiated at different excitation wavelengths.

### MRSA infection mouse model

The mice were s.c. injected with LysGH15 (50 μg). After 3 weeks, when the anti-LysGH15 antibodies reached the highest titre, the mice were challenged intravenously with YB57 MRSA (1 × 10^10^ CFU) as described above. Subsequently, a single dose of LysGH15 (50 μg) was administered intravenously at 1 h after the bacterial challenge^43^. The control group was treated with an equal amount of buffer under the same conditions. At intervals, the bacterial counts were determined from 10 μL of peripheral blood samples obtained from the caudal veins of mice treated with either LysGH15 or buffer^16^.

### Toxicity assays

BALB/c mice immunized with LysGH15 (50 μg) for 3 weeks were intravenously injected one time with 10 mg of LysGH15 (n = 6). Their aspect and behaviour were examined daily for 10 days. Health scores were determined using a method modified from a previous report^34^. A score of 5 indicated normal health and an unremarkable condition. Slight illness was defined as decreased physical activity and ruffled fur and was scored as 4. Moderate illness was defined as lethargy and a hunched back and was scored as 3. Severe illness was defined as the aforementioned signs plus exudative accumulation around partially closed eyes and was scored as 2. A moribund state was scored as 1, and death was scored as 0. Each dot indicates the state of health of a single mouse. After 10 days, the high-dose group and the immunized group were euthanized through intraperitoneal injection with ketamine and xylazine and used for histopathology analysis. The organs, including the heart, liver, spleen, lung, kidney, and colon, were removed and immediately placed in 4% formalin. The formalin-fixed tissues were processed and stained with haematoxylin and eosin (H&E) and toluidine blue staining using a routine staining procedure and subsequently analysed using microscopy^44^.

### Data analysis

GraphPad Prism 5 (GraphPad Software, Inc., CA, USA) was utilized to analyse the data measured using ELISA; SPSS version 13.0 (SPSS, Inc., Chicago, IL, USA) was used for the statistical analysis of other experimental data using one-way analysis of variance. A P-value < 0.05 was considered statistically significant. Error bars represent the standard deviation of the mean.

## Additional Information

**How to cite this article**: Zhang, L. *et al*. LysGH15 kills *Staphylococcus aureus* without being affected by the humoral immune response or inducing inflammation. *Sci. Rep.* **6**, 29344; doi: 10.1038/srep29344 (2016).
